# Prognostic Benefit of Segmentectomy for Patients with Low Muscle Mass in Early-Stage Lung Cancer

**DOI:** 10.1245/s10434-024-16384-5

**Published:** 2025-04-24

**Authors:** Daisuke Ueda, Takahiro Mimae, Atsushi Kamigaichi, Norifumi Tsubokawa, Yoshihiro Miyata, Kenichi Yoshimura, Morihito Okada

**Affiliations:** 1 Department of Surgical Oncology, Research Institute for Radiation Biology and Medicine, Hiroshima University, Hiroshima, Japan; 2 Medical Center for Clinical and Translational Research, Hiroshima University Hospital, Hiroshima, Japan; 3 Department of Biostatistics and Health Data Science, Nagoya City University School of Medical Science, Nagoya, Japan

**Keywords:** Lobectomy, Muscle mass, Non-small cell lung cancer, Sarcopenia, Segmentectomy

## Abstract

**Background:**

Although segmentectomy is less invasive than lobectomy, little is known about the effect of these procedures on postoperative outcomes of patients with low muscle mass. This study examined the postoperative prognosis of patients with non-small cell lung cancer according to the surgical procedure type and preoperative muscle mass.

**Methods:**

This study retrospectively reviewed data of patients who underwent lobectomy or segmentectomy for early-stage non-small cell lung cancer with ground glass opacity-dominant tumor up to 3 cm in size or solid-dominant tumor up to 2 cm in size between 2010 and 2020. The preoperative muscle mass was evaluated based on the height-adjusted erector spinae muscle mass on preoperative chest computed tomography images. The overall survival was compared between patients with low and high muscle mass in the lobectomy and segmentectomy groups. Multivariable analyses were performed to identify prognostic factors for overall survival.

**Results:**

The study enrolled 371 patients: 162 in the lobectomy group and 209 in the segmentectomy group. The 5-year overall survival was significantly poorer for the patients with low muscle mass than for those with high muscle mass in the lobectomy group (83.9 % vs. 91.9 %; *p* = 0.018), whereas no significant difference was observed in the segmentectomy group (93.5 % vs. 94.9 %; *p =* 0.54). In the several multivariable models, low muscle mass was an independent prognostic factor in the lobectomy group (hazard ratio [HR], range, 2.3−2.4; *p* value range, 0.027−0.042) but not in the segmentectomy group (HR range, 1.6−2.0; *p* value range, 0.19−0.36).

**Conclusions:**

Segmentectomy should be actively considered for patients with low muscle mass because of its lower invasiveness.

**Supplementary Information:**

The online version contains supplementary material available at 10.1245/s10434-024-16384-5.

Sublobar resection is increasingly performed for peripheral small-size lung cancer.^1,2^Among sublobar resection techniques, segmentectomy has proved to be a good treatment option because it achieves better local control than wedge resections and is less invasive than lobectomy.^3,4^ A large prospective randomized trial demonstrated that segmentectomy was superior to lobectomy in terms of overall survival (OS) for patients with small-size non-small cell lung cancer (NSCLC).^5^ One possible reason for that finding is the lower invasiveness of segmentectomy compared with lobectomy.^5^

Sarcopenia, or low muscle mass, has become a widely recognized risk factor for poorer prognosis in many benign and malignant conditions.^6^ Preoperative sarcopenia has been associated with postoperative prognoses in many types of cancer. For patients with lung cancer, preoperative sarcopenia or low muscle mass is a prognostic factor for poor postoperative OS.^7,8^ In our previous study focused on the erector spinae muscles (ESMs), antigravity muscles that reflect physical activity, low preoperative ESM mass was associated with poor postoperative OS, mostly because of increased non-lung cancer-related mortality.^8,9^

The aforementioned factors indicate that patients with low muscle mass may be more vulnerable to surgical intervention than those with normal/high muscle mass and might benefit more from segmentectomies than from lobectomies in terms of postoperative general condition. Nevertheless, studies supporting this observation are scarce. Therefore, we investigated the impact of preoperative ESM mass and surgical procedure types on the postoperative prognosis of patients with completely resected early-stage NSCLC.

## Methods

### Ethical Statement

The Institutional Review Board of Hiroshima University Hospital, Hiroshima, Japan, approved this study on 25 June 2021 (approval no. Hi-E-3930). The need for informed consent was waived because of the retrospective study design.

### Study Design and Population

**Fig. 1:**
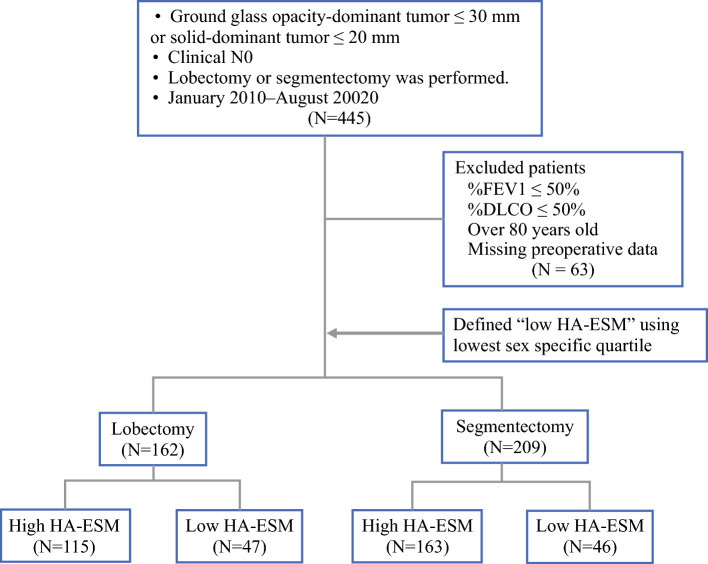
Study flowchart. DLCO, diffusing lung capacity for carbon monoxide; FEV, forced expiratory volume; HA-ESM, height-adjusted erector spinae muscle

We retrospectively reviewed the records of consecutive patients with clinical stage IA NSCLC who underwent lobectomies or segmentectomies as curative surgery at Hiroshima University Hospital between January 2010 and August 2020. Because our surgical indication for intentional segmentectomy was clinical stage 0 or IA cancer with a ground-glass opacity-dominant tumor up to 3 cm in size or a solid-dominant tumor up to 2 cm in size on preoperative high-resolution computed tomography (CT), patients with solid tumors exceeding 2 cm in size were excluded.^10^ Patients with a forced expiratory volume (FEV) in the first second (FEV1) of ≤50 % or diffusing lung capacity for carbon monoxide (DLCO) of ≤50 % were also excluded as high-risk patients per the major criteria in the ACOSOG Z4032 study.^11^ Patients older than 80 years, those receiving neoadjuvant chemotherapy, and those with histopathologically revealed R1 resections also were excluded (Fig. 1).

The pathologic diagnosis was determined according to the World Health Organization 2015 classification.^12^ Staging was performed according to the International Association for the Study of Lung Cancer’s eighth tumor-node-metastasis (TNM) classification.^13^ The 18F-fluorodeoxyglucose-positron emission tomography/CT scans were evaluated visually using the Deauville criteria, as previously reported.^14,15^

### Classification of Muscle Mass

Preoperative muscle mass was evaluated based on the height-adjusted ESM mass (HA-ESM) on preoperative chest CT images. The details regarding the calculation of HA-ESM are described in our previous study.^8^ Tanimura’s method of measurement was followed.^16^ Briefly, measurements were performed bilaterally at the level of the lower margin of the 12th thoracic vertebra on preoperative CT images (Fig. S1). All ESM regions identified in the mediastinal setting were manually encircled, and the ESM area was automatically calculated. The ESM area was normalized for patient height (cm^2^/m^2^; ESM area/height × height). The normalized score was called the HA-ESM. A low HA-ESM was defined as an HA-ESM value less than the sex-specific lowest quartile of HA-ESM. Patients other than those with low HA-ESM were classified as having high HA-ESM, which had been named high and moderate ESM in our previous study, and were renamed in this study for a simple description.

### Statistical Analysis

Overall survival was defined as the period from the date of surgery to the date of the last follow-up visit or death from any cause and calculated using the log-rank test. According to the cause of death, patients were divided into two groups: lung cancer-related and non-lung cancer-related death. The cumulative mortality rates in these two groups were calculated using Gray’s test. The significance of frequencies was evaluated using the chi-square test. Continuous and ordinal variables were compared using the Mann–Whitney *U* test. Cox proportional hazard regression analysis was applied to evaluate the impact of each variable on the OS.

The propensity score, reflecting the probability of segmentectomy, was derived from a multivariate logistic regression model. Inverse probability of treatment weighting, with average treatment effect based on the propensity score, was performed to adjust for patients’ backgrounds between groups when the prognoses of procedures were compared. Propensity score-matching was used to balance the preoperative baseline characteristics when the prognoses of patients with high and low HA-ESMs were compared.

Statistical analyses were performed using EZR version 1.33 (Saitama Medical Center, Jichi Medical University, Shimotsuke, Tochigi, Japan), a graphic user interface for R version 3.4.4 (The R Foundation for Statistical Computing, Vienna, Austria).^17^

## Results

### Patient Characteristics

Among the 371 patients included in the review, 162 underwent lobectomy, and 209 underwent segmentectomy. The median follow-up period was 7.15 years. The cutoff value for the HA-ESM was 11.14 for males and 9.90 for females. Of the 371 patients, 93 had low HA-ESMs. All the patients were east Asian.

### Impact of the Surgical Procedure on OS According to HA-ESM Status

Patients who underwent right middle lobectomies were excluded because segmentectomies were not usually performed in this lobe. In the high HA-ESM group, lobectomy was significantly associated with a higher frequency of smoking (*p =* 0.022), higher Deauville score (*p* < 0.001), larger whole tumor size (*p* < 0.001), larger solid tumor size (*p* < 0.001), and higher clinical stage, 1 (*p* < 0.001). In the low HA-ESM group, lobectomy was significantly associated with larger solid tumor size (*p =* 0.002) and higher clinical stage (*p =* 0.008) (Table S1).

After weight adjustment for the variables of age, sex, smoking status, resection of upper or lower lobe, Charlson Comorbidity Index (>2), Deauville score (3–5), and clinical stage, the procedure type (segmentectomy) was not an independent prognostic factor for OS in either the high HA-ESM group (hazard ratio [HR] 0.85; 95% confidence interval [CI] 0.39–1.88; *p =* 0.69) or the low HA-ESM group (HR 0.72; 95% CI 0.23–2.92; *p =* 0.56).

### Impact of HA-ESM Status on OS According to Surgical Procedure

**Fig. 2:**
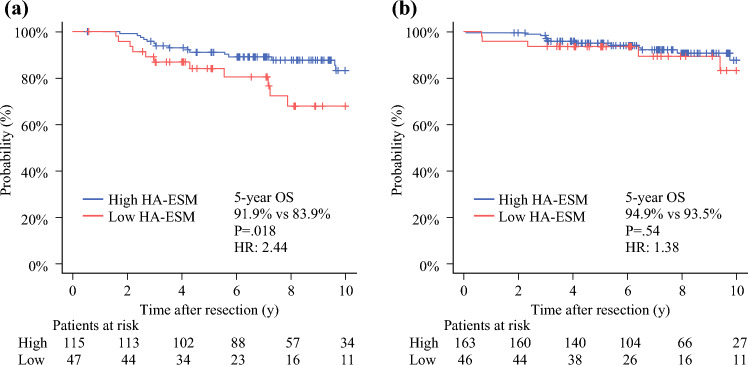
Overall survival according to height-adjusted erector spinae muscle mass in the (**a**) lobectomy and (**b**) segmentectomy groups. High HA-ESM is indicated by the blue line and low HA-ESM by the red line. HA-ESM, height-adjusted erector spinae muscle; HR, hazard ratio; OS, overall survival

Low HA-ESM was significantly associated with lower %DLCO in the lobectomy (*p =* 0.020) and segmentectomy (*p =* 0.002) groups and with a higher frequency of lymphovascular invasion in the segmentectomy group (*p =* 0.028). No significant differences were observed in pathologic tumor size or stage between the groups (Table 1). In the lobectomy group, the patients with low HA-ESMs had significantly poorer 5-year OS than those with high HA-ESMs (83.9 % vs. 91.9 %; HR 2.44; 95% CI 1.14–5.22; *p =* 0.018; Fig. 2A). In contrast, no significant difference in the 5-year OS was observed in the segmentectomy group (93.5 % vs. 94.9 %; HR 1.38; 95% CI 0.49–3.87; *p =* 0.54; Fig. 2B).

**Table 1:** Baseline patient characteristics

Factor		Lobectomy		*p* Value	Segmentectomy		*p* Value
		High HA-ESM	Low HA-ESM		High HA-ESM	Low HA-ESM	
		(*n* = 115 *n* (%)	(*n* = 47) *n* (%)		(*n* = 163) *n* (%)	(*n* = 46) *n* (%)	
Age (years)	Median	64	72	0.06	68	66	0.51
	IQR	60−72	63.5−75		61.5−72	61−72	
Sex	Male	68 (59.1)	26 (55.3)	0.73	81 (49.7)	24 (52.2)	0.87
Smoking status	Smoker	68 (59.1)	25 (53.2)	0.49	74 (45.4)	27 (58.7)	0.13
FEV1	≥70	92 (80.0)	39 (83.0)	0.83	130 (79.8)	32 (69.6)	0.16
Charlson Comorbidity Index	Median	2	2	0.84	2	2	0.35
	IQR	2−3	2−3.5		2−4	2−4	
Hypertension	Yes	25 (21.7)	16 (34.0)	0.11	45 (27.6)	8 (17.4)	0.183
Myocardial infarction or heart failure	Yes	6 (5.2)	3 (6.4)	0.72	3 (1.8)	0 (0)	1
%FEV1	Median	96.9	98.9	0.74	86.8	93.5	0.45
	IQR	83.8−109.8	85.8−110.8		96.5−107.9	85.0−103	
%DLCO	Median	82.7	76.9	0.020	85.4	77.6	0.002
	IQR	72.2−93.9	66.8−86.6		75.3−94.5	70.9−83.1	
Operation side	Right	89 (77.4)	37 (78.7)	1	80 (49.1)	18 (39.1)	0.25
Operation lobe	Upper	66 (57.4)	27 (57.4)	0.31	95 (58.3)	29 (63.0)	0.84
	Lower	33 (28.7)	9 (19.1)		66 (40.5)	17 (37.0)	
	Middle	13 (11.3)	10 (21.3)		0	0	
	Bilobe	3 (2.6)	1 (2.1)		2 (1.2)	0	
Deauville score	Median	2	2	0.9	2	2	0.64
	IQR	2−4	2−4		2−2.5	1−3	
Whole tumor size on CT (mm)		18	18	0.72	15	15	0.4
	IQR	15−22	15−22		12−19	13−20	
Solid tumor size on CT (mm)		14	14	0.95	10	10	0.32
	IQR	10−17	10−17		6−14	5−13	
Clinical stage	IA1	33 (28.7)	12 (25.5)	0.85	87 (53.4)	26 (56.5)	0.74
	IA2	82 (71.3)	35 (74.5)		76 (46.6)	20 (43.5)	
Pathological size (mm)	Median	17	17	0.86	12	15	0.93
	IQR	15−22	14−24		15−20	13−20	
Pathologic invasive size (mm)	Median	13	15	0.6	6	10	0.86
	IQR	8−17	9−18		9−15	6−14	
Pathologic stage	0	4 (3.5)	3 (6.4)	0.45	19 (11.7)	8 (17.4)	0.89
	IA1	39 (33.9)	12 (25.5)		72 (44.2)	18 (39.1)	
	IA2	40 (34.8)	18 (38.3)		49 (30.1)	16 (34.8)	
	IA3	5 (4.3)	5 (10.6)		6 (3.7)	1 (2.2)	
	IB	12 (10.4)	4 (8.5)		11 (6.7)	3 (6.5)	
	IIB	10 (8.7)	5 (10.6)		4 (2.5)	0	
	IIIA	5 (4.3)	0		2 (1.2)	0	
Histologic type	Adenocarcinoma	99 (86.1)	42 (89.4)	0.8	152 (93.3)	41 (89.1)	0.35
	Other	16 (13.9)	5 (10.6)		11 (6.7)	5 (10.9)	
Pleural invasion	Yes	15 (13.0)	6 (12.8)	1	12 (7.4)	2 (4.3)	0.74
Lymphovascular invasion	Yes	36 (31.3)	9 (19.1)	0.13	24 (14.7)	14 (30.4 %)	0.03

*HA-ESM* Height-adjusted erector spinae muscle, *IQR* Interquartile range, *FEV* Forced expiratory volume, *DLCO* Diffusing lung capacity for carbon monoxide, *CT* Computed tomography

**Fig. 3:**
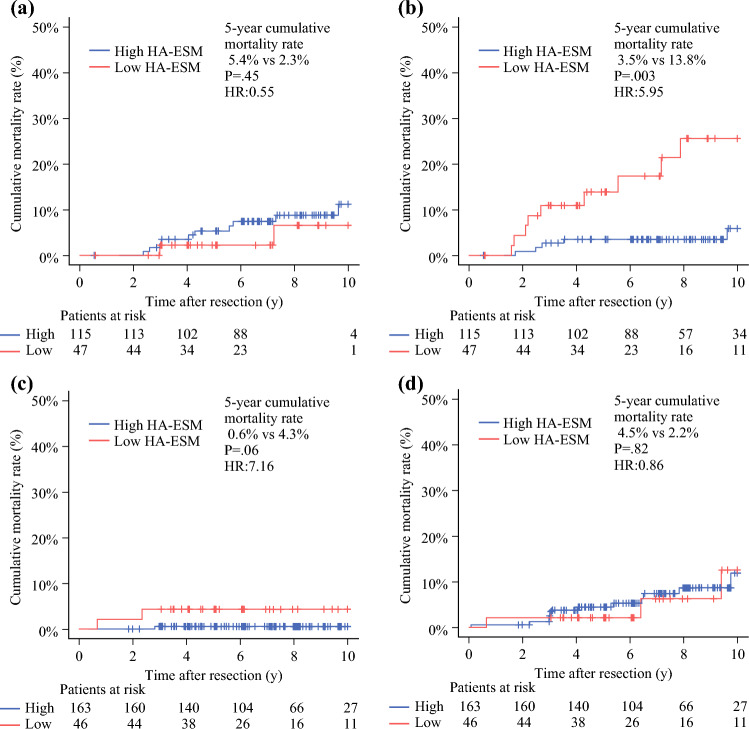
Cancer- and non-cancer-related mortality according to height-adjusted erector spinae muscle mass. Cumulative mortality curves show (**a**) cancer-related and (**b**) non-cancer-related mortality in the lobectomy group and of (**c**) cancer-related and (**d**) non-cancer-related mortality in the segmentectomy group. High HA-ESM is indicated by the blue line and low HA-ESM by the red line. HA-ESM, height-adjusted erector spinae muscle; HR, hazard ratio

Next, we compared the 5-year cumulative lung cancer- and non-lung cancer-related mortality in both groups. In the lobectomy group, no significant difference was found between high and low HA-ESM in lung cancer-related mortality (5.4 % vs. 2.3 %; HR 0.55; 95% CI 0.12–2.49; *p =* 0.44; Fig. 3A). However, non-lung cancer-related mortality was significantly higher in the patients with low HA-ESMs than in those with high HA-ESMs (﻿3.5% vs. 13.8% ; HR 5.95; 95% CI 2.02–17.53; *p =* 0.003; Fig. 3B). In the segmentectomy group, no significant differences were noted in either lung cancer-related (0.6 % vs. 4.3 %; HR 7.16; 95% CI 0.66–77.56; *p =* 0.06; Fig. 3C) or non-lung cancer-related mortality (4.5 % vs. 2.2 %; HR 0.86; 95% CI 0.25–2.98; *p =* 0.82; Fig. 3D). For non-lung cancer-related mortality, the common causes of death in both groups were respiratory disease (7 cases), cardiovascular diseases (6 cases), and second cancer (10 cases). In the lobectomy group, mortality due to respiratory and cardiovascular diseases was higher among the patients with low HA-ESMs than among those with high HA-ESMs (1.8% vs. 9.5%; HR 5.49; 95% CI 1.02–29.52; *p =* 0.028), whereas such a trend was not observed in the segmentectomy group (1.4% vs. 0%; HR 1.43; 95% CI 0.30–6.78; *p =* 0.67; Fig. S2).

### Prognostic Factors for OS According to Surgical Procedure

The lobectomy group had 27 OS events, and the segmentectomy group had 18 OS events. To confirm whether other variables were confounding factors, we performed multivariate analyses in several models. In the lobectomy group, the HRs of the HA-ESMs were similar (2.265−2.415), and all *p* values were below 0.05 in every multivariate model (Table 2). In contrast, the HRs in the segmentectomy group ranged from 1.624 to 2.025, and all *p* values were higher than 0.05 (0.19−0.36) (Table 3). Therefore, low HA-ESM was an independent prognostic factor in the lobectomy group but not in the segmentectomy group.

**Table 2:** Uni- and multivariable analyses of overall survival in the lobectomy group

	Univariable analysis		Multivariate analysis		Multivariate analysis		Multivariate analysis		Multivariate analysis	
			Model 1		Model 2		Model 3		Model 4	
Factor	HR (95% CI)	*p* Value	HR (95% CI)	*p* Value	HR (95% CI)	*p* Value	HR (95% CI)	*p* Value	HR (95% CI)	*p* Value
Age (years)	1.104 (1.045–1.167)	<0.001	1.099 (1.0380–1.163)	0.001	1.092 (1.030–1.158)	0.003	1.097 (1.036–1.162)	0.002	1.095 (1.035–1.158)	0.002
Sex (male)	2.488 (1.049–5.899)	0.039	1.091 (.3511–3.391)	0.88	–	–	–	–	–	–
Smoking status (smoker)	4.29 (1.613–11.41)	0.004	4.596 (1.249–16.910)	0.022	4.566 (1.663–12.530)	0.003	3.687 (1.295–10.500)	0.015	4.734 (1.730–12.950)	0.002
Charlson Comorbidity Index (>3)	2.419 (1.127–5.19)	0.023	–	–	1.449 (.6538–3.213)	0.36	–	–	–	–
Deauville score (>3)	3.487 (1.523–7.986)	0.003	–	–	–		2.241 (.9455–5.312)	0.07	–	–
Clinical stage (IA2)	3.311 (.9965–11.0)	0.051	–	–	–	–	–	–	2.252 (.6706–7.565)	0.20
HA-ESM (low)	2.439 (1.14–5.22)	0.022	2.415 (1.104–5.285)	0.027	2.375 (1.084–5.201)	0.031	2.286 (1.044–5.007)	0.039	2.265 (1.031–4.973)	0.042

HR, hazard ratio; CI, confidence interval; HA-ESM, height-adjusted erector spinae muscle

**Table 3:** Uni- and multivariable analyses for overall survival in the segmentectomy group

	Univariable analysis		Multivariate analysis		Multivariate analysis		Multivariate analysis		Multivariate analysis		Multivariate analysis	
			Model 1		Model 2		Model 3		Model 4		Model 5	
Factor	HR (95% CI)	*p* Value	HR (95% CI)	*p* Value	HR (95% CI)	*p* Value	HR (95% CI)	*p* Value	HR (95% CI)	*p* Value	HR (95% CI)	*p* Value
Age (years)	1.142 (1.049–1.242)	0.002	1.145 (1.051–1.248)	0.002	1.175 (1.071–1.289)	0.001	1.134 (1.042–1.234)	0.003	1.143 (1.049–1.246)	0.002	1.141 (1.045–1.245)	0.003
Sex (male)	8.086 (1.859–35.17)	0.005	7.667 (1.762–33.370)	0.007	–	–	–	–	–	–	–	–
Smoking status (smoker)	5.954 (1.722–20.58)	0.005	–	–	6.825 (1.954–23.840)	0.003	–	–	–	–	–	–
Charlson Comorbidity Index (>3)	3.978 (1.408–11.24)	0.009	–	–	–	–	3.429 (1.188–9.893)	0.023	–	–	–	–
Deauville score (>3)	2.735 (1.073–6.967	0.035	–	–	–	–	–	–	2.355 (.9245–6.000)	0.07	–	–
Clinical stage (IA2)	4.176 (1.374–12.69)	0.011	–	–	–	–	–	–	–	–	3.433 (1.127–10.460)	0.030
HA-ESM (low)	1.378 (.490–3.873)	0.54	1.624 (.571–4.621)	0.36	1.680 (.582–4.853)	0.34	2.025 (.707–5.799)	0.19	1.735 (.604–4.984)	0.31	1.819 (.641–5.166)	0.26

HR, hazard ratio; CI, confidence interval; HA-ESM, height-adjusted erector spinae muscle

For propensity score-matching, the variables included age, sex, smoking status, Charlson Comorbidity Index (>2), respiratory function (%FEV >95% and %DLCO >85), clinical stage, and Deauville score (>2). The cutoff value of %FEV and %DLCO was determined in reference to their median values (96.9 % and 82.0 %, respectively).

**Fig. 4:**
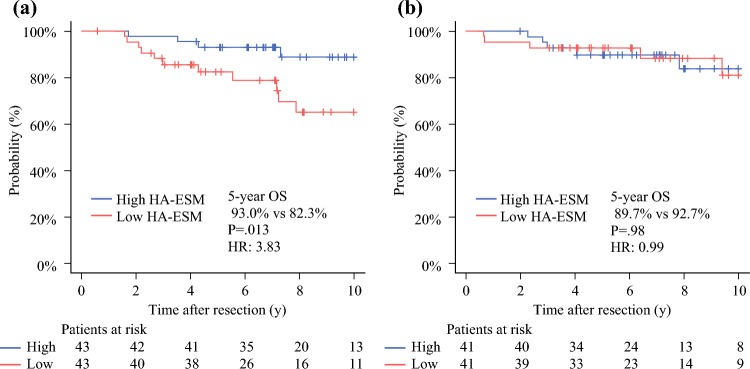
Overall survival according to height-adjusted erector spinae muscle mass in the (**a**) lobectomy and (**b**) segmentectomy groups after propensity score-matching. High HA-ESM is indicated by the blue line and low HA-ESM by the red line. HA-ESM, height-adjusted erector spinae muscle; HR, hazard ratio

After matching, no significant differences in clinical factors were observed between the groups (Table S2). In the lobectomy group, the patients with low HA-ESMs had significantly poorer OS rates than those with high HA-ESMs (82.3 % vs. 93.0 %; HR 3.83; 95% CI 1.23–11.89; *p =* 0.013; Fig. 4A), but no significant difference was observed in the segmentectomy group (92.7 % vs. 89.7 %; HR 0.99; 95% CI 0.29–3.41; *p =* 0.98; Fig. 4B).

## Discussion

The patients in the current study with low HA-ESMs had significantly poorer OS rates and higher non-lung cancer-related mortality rates than those with high HA-ESMs in the lobectomy group, whereas no significant differences were found in the segmentectomy group. A low HA-ESM was an independent prognostic factor for OS in the lobectomy group but not in the segmentectomy group.

In the JCOG0802/WJOG4607L randomized trial, segmentectomy was superior to lobectomy in terms of OS for patients with small and peripheral NSCLC.^5^ It was hypothesized that segmentectomy, which preserves more lung parenchyma, may have allowed more extensive treatment of other diseases. Based on this concept, segmentectomy may be more beneficial for vulnerable patients with lower functional reserves than for healthy patients. In fact, several studies have indicated that patients with lower functional reserves benefit from sublobar resections. Mimae et al.^18^ demonstrated that older patients (age ≥80 years) had poorer prognoses than younger patients after wedge resections for early-stage NSCLC and that wedge resection could reduce mortality due to other causes. In the report, the author mentioned that segmentectomy for octogenarians did not reduce non-cancer-related mortality, and we performed the analysis of patients other than octogenarians. Isaka et al.^19^ reported that patients with high comorbidity indexes who underwent segmentectomies had better prognoses than those who underwent lobectomies because of reduced cumulative mortality due to respiratory diseases and other cancers. However, only one study has considered the clinical benefits of sublobar resections for patients with sarcopenia.^20^

To the best of our knowledge, the current study is the first to report that segmentectomy is superior to lobectomy for patients with low muscle mass, which includes many patients with sarcopenia. Because the poorer OS of patients with low HA-ESMs in the lobectomy group was caused by an increase in non-lung cancer-related mortality, the benefit of segmentectomy may be due to reduced non-lung cancer-related mortality.

In terms of lower functional reserves, there may be an overlap with old age, low respiratory function, high comorbidity rate, and low muscle mass. In the lobectomy group, the patients with low muscle mass had significantly worse %DLCO and tended to be older. However, after propensity-matching adjustments for age, respiratory function, and comorbidities, low muscle mass was a prognostic factor in the lobectomy group but not in the segmentectomy group. Although the lobectomy and segmentectomy groups comprised different cohorts, the patients with low muscle mass were more likely to benefit from segmentectomy than from lobectomy.

The reason for the aforementioned benefits of segmentectomy compared with lobectomy remains unclear. Previous studies have suggested that segmentectomy preserves respiratory function better than lobectomy.^5,21^ Theoretically, the load to the right heart is thought to be larger after lobectomy than after segmentectomy.^22^ In our study, preserving respiratory function and reducing the load on the right heart in the segmentectomy group may have contributed to an improved prognosis for the patients with low muscle mass. In fact, our results showed that cumulative mortality due to respiratory and cardiovascular diseases was significantly higher for the patients with low HA-ESMs than for those with high HA-ESMs in the lobectomy group.

Although current evidence supports resistance exercises for sarcopenia treatment, little evidence exists to show that treatment can improve postoperative prognoses.^23^ Nonetheless, a treatment with reduced invasion to the lung parenchyma might be a better option for patients with lung diseases and sarcopenia requiring lung resections.

Our study had several limitations. First, it was a retrospective single-center study. Therefore, selection bias likely existed due to the surgeons’ patient selection process. Additionally, the cutoff value was calculated at our institution, and its appropriateness could not be confirmed. Future multicenter studies are warranted to reduce selection bias and set an appropriate cutoff value.

Second, in this study, we did not adopt the sarcopenia definition per the European Working Group on Sarcopenia in Older People (EWGSOP2). The guidelines demonstrate that probable sarcopenia is first identified by low muscle strength and next confirmed by low muscle quantity.^6^ Instead, we used HA-ESM as an indicator of muscle loss because we could not obtain data on muscle strength. Although using CT imaging is thought to be acceptable in cancer research, muscle strength is necessary for formal diagnosis of sarcopenia. A prospective observational study including muscle strength evaluation is needed for further understanding.

Third, due to the small number of patients and differences in patient backgrounds, we could not perform a direct comparison between lobectomy and segmentectomy.

In conclusion, patients with low muscle mass deemed to be at risk are more likely to benefit from segmentectomies than from lobectomies in terms of mortality due to other causes. When feasible, surgical procedures should be selected considering not only the patient’s age, respiratory function, and morbidities, but also the patient’s muscle mass.

## Supplementary Information

Below is the link to the electronic supplementary material.

Fig. S1 Representative preoperative computed tomography image showing the erector spinae muscle mass measurement method. The measurement was performed bilaterally at the level of the lower margin of the 12th thoracic vertebra on axial scans

Fig. S2 Mortality due to respiratory and cardiovascular diseases according to height-adjusted erector spinae muscle mass in the (a) lobectomy and (b) segmentectomy groups. HA-ESM, height-adjusted erector spinae muscle; HR, hazard ratio

Supplementary file3 (DOCX 32 KB)
