# Supplementary figures and images for: Prognostic Benefit of Segmentectomy for Patients with Low Muscle Mass in Early-Stage Lung Cancer
Source: Ann Surg Oncol. 2025 Apr 24;32(7):4660–9. doi: 10.1245/s10434-024-16384-5 (PMC12130164; doi:10.1245/s10434-024-16384-5)

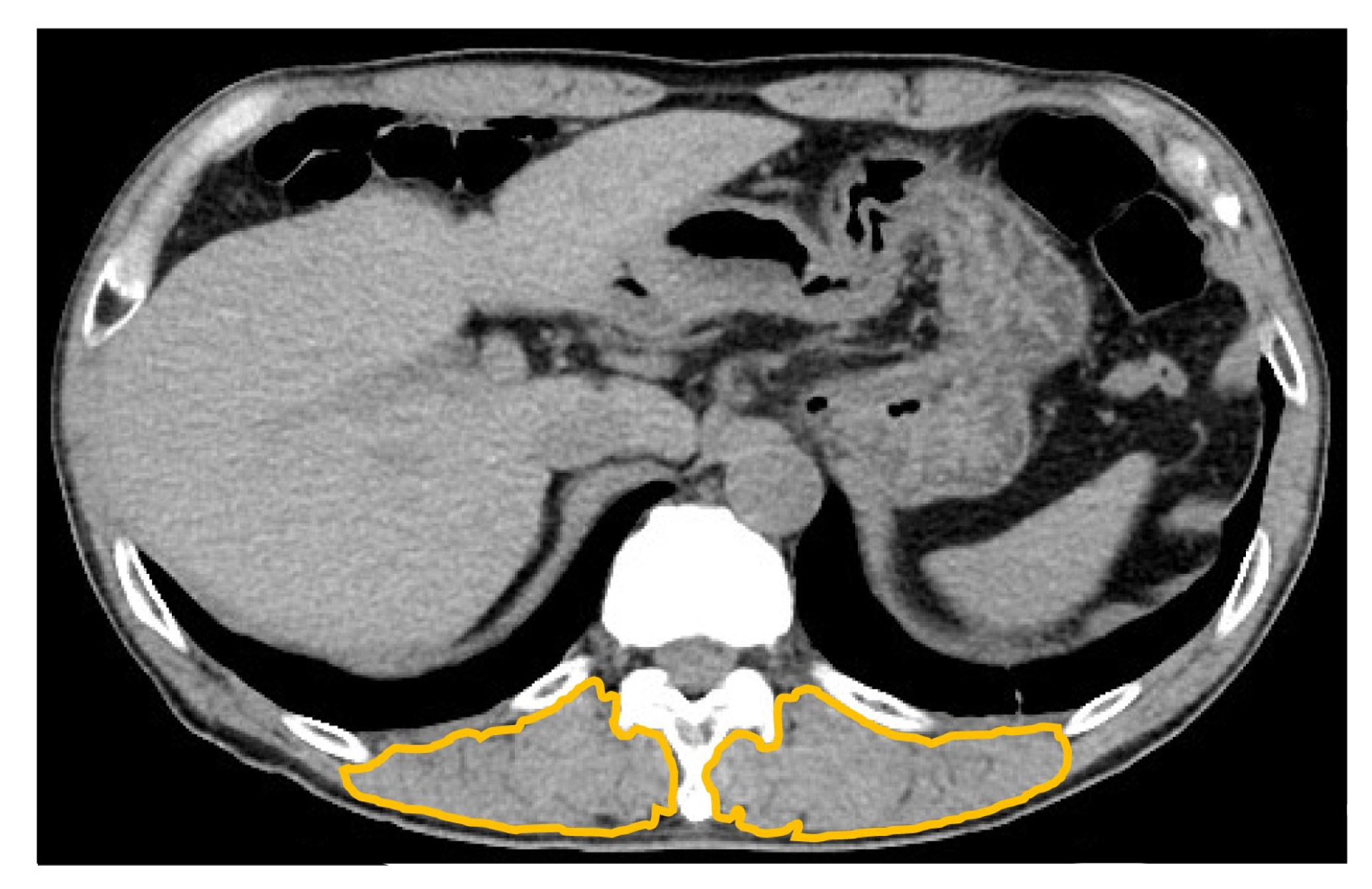

Supplement: Supplementary file 1 — Fig. S1 Representative preoperative computed tomography image showing the erector spinae muscle mass measurement method. The measurement was performed bilaterally at the level of the lower margin of the 12th thoracic vertebra on axial scans [file 10434_2024_16384_MOESM1_ESM.tif]

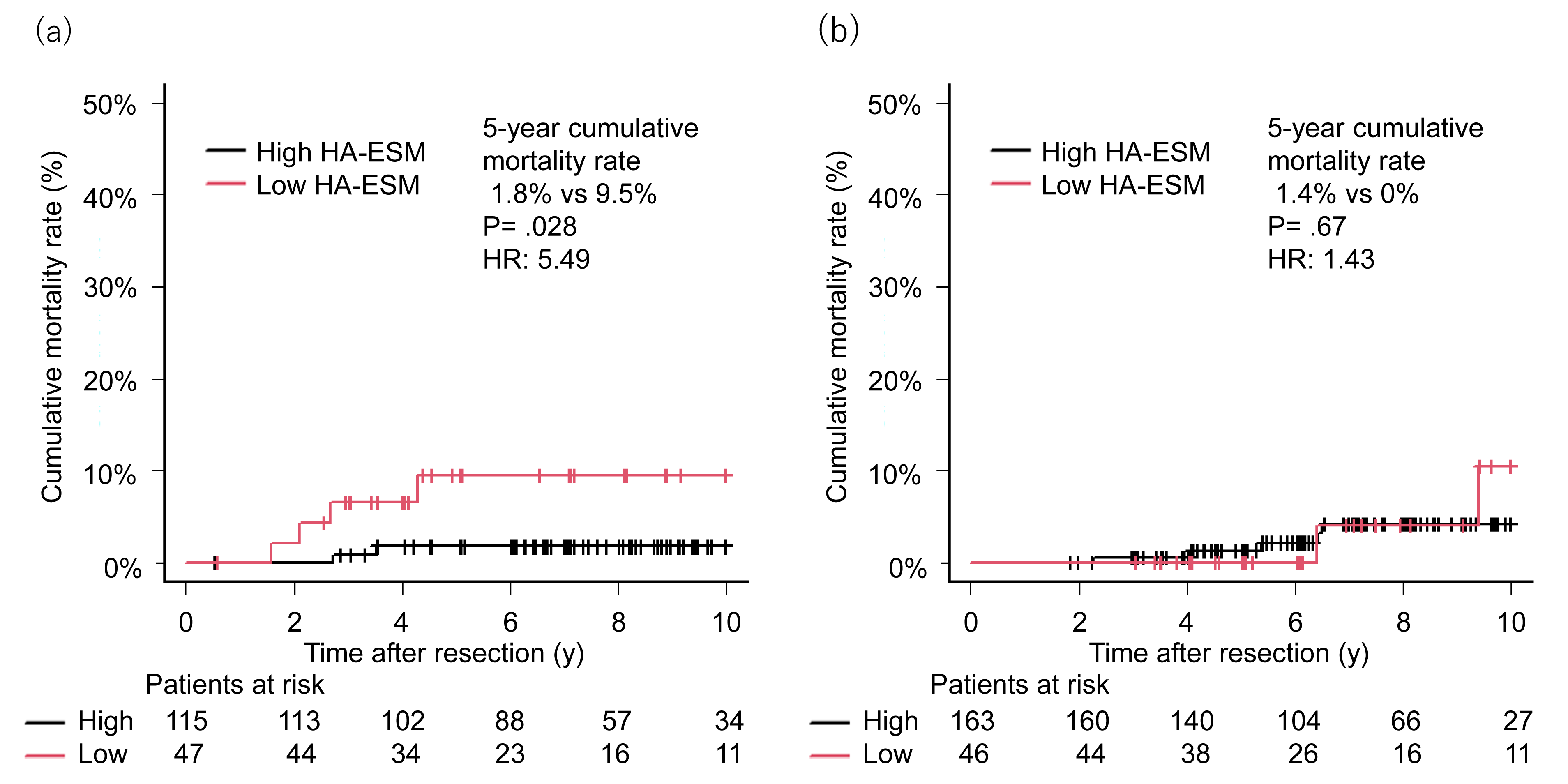

Supplement: Supplementary file 2 — Fig. S2 Mortality due to respiratory and cardiovascular diseases according to height-adjusted erector spinae muscle mass in the (a) lobectomy and (b) segmentectomy groups. HA-ESM, height-adjusted erector spinae muscle; HR, hazard ratio [file 10434_2024_16384_MOESM2_ESM.tif]
