# Supplementary material for: Prognostic Benefit of Segmentectomy for Patients with Low Muscle Mass in Early-Stage Lung Cancer
Source: Ann Surg Oncol. 2025 Apr 24;32(7):4660–9. doi: 10.1245/s10434-024-16384-5 (PMC12130164; doi:10.1245/s10434-024-16384-5)
Supplement: Supplementary file 3 — Supplementary file3 (DOCX 32 KB) [file 10434_2024_16384_MOESM3_ESM.docx]

Supplementary Table 1. Baseline characteristics of patients according to HA-ESM status.

| Factor |  | High HA–ESM | |  | Low HA–ESM | |  |
| --- | --- | --- | --- | --- | --- | --- | --- |
|  |  | Lobectomy | Segmentectomy |  | Lobectomy | Segmentectomy |  |
|  |  | n=100 | n=163 | *P* | n=37 | n=46 | *P* |
| Age, years | Median | 64 | 68 | .19 | 75 | 66 | .39 |
|  | IQR | 60–71 | 62–72 |  | 62–75 | 61–72 |  |
| Sex, n (%) | Male | 59 (59.0%) | 81 (49.7%) | .16 | 20 (54.1%) | 24 (52.2%) | 1 |
| Smoking status, n (%) | Smoker | 60 (60.0%) | 74 (45.4%) | .023 | 19 (51.4%) | 27 (58.7%) | .52 |
| FEV1, n (%) | ≥70 | 81 (81.0%) | 130 (79.8%) | .87 | 29 (78.4%) | 32 (69.6%) | .47 |
| Charlson Comorbidity Index | Median | 2 | 2 | .054 | 2 | 2 | .65 |
|  | IQR | 2–3 | 2–4 |  | 2–3 | 2–4 |  |
| %FEV1 | Median | 96.5 | 96.5 | .55 | 96.7 | 93.5 | .68 |
|  | IQR | 82.2–108.9 | 86.8–107.9 |  | 85.1–105.8 | 85.0–103 |  |
| %DLCO | Median | 82.3 | 85.4 | .43 | 78.3 | 77.6 | .54 |
|  | IQR | 72.2–94.5 | 75.3–94.5 |  | 68–87.3 | 70.9–83.1 |  |
| Operation side, n (%) | Right | 74 (74.0%) | 80 (49.1%) | <.001 | 10 (27.0%) | 18 (39.1%) | .004 |
|  | Left | 26 (26.0%) | 83 (50.9%) |  | 27 (73.0) | 28 (60.9%) |  |
| Operation lobe, n (%) | Upper | 66 (66.0%) | 95 (58.3%) | .45 | 27 (73.0%) | 29 (63.0%) | .24 |
|  | Lower | 33 (33.0%) | 66 (40.5%) |  | 9 (24.3%) | 17 (37.0%) |  |
|  | Middle | 0 | 0 |  | 0 | 0 |  |
|  | Bilobe | 1 (1.0%) | 2 (1.2%) |  | 1 (2.7%) | 0 |  |
| Deauville score | Median | 3 | 2 | <.001 | 2 | 2 | .06 |
|  | IQR | 2–3 | 2–2.5 |  | 2–4 | 1–3 |  |
| Nodule diameter on CT (mm) | | 18 | 15 | <.001 | 18 | 15 | .15 |
|  | IQR | 15–22 | 12–19 |  | 15–21 | 13–20 |  |
| Solid tumor size on CT (mm) | | 15 | 10 | <.001 | 12 | 10 | .002 |
|  | IQR | 9–17 | 6–14 |  | 10–17 | 5–13 |  |
| Clinical stage, n (%) | IA1 | 31 (31.0%) | 87 (53.4%) | <.001 | 10 (27.0%) | 26 (56.5%) | .008 |
|  | IA2 | 69 (69.0%) | 76 (46.6%) |  | 27 (73.0%) | 20 (43.5%) |  |
| Pathological size (mm) | Median | 18 | 15 | <.001 | 15 | 15 | .43 |
|  | IQR | 15–22 | 12–20 |  | 13–21 | 13–20 |  |
| Pathological invasion size (mm) | Median | 13 | 9 | <.001 | 13 | 10 | .14 |
|  | IQR | 8–18 | 6–15 |  | 8–18 | 6–14 |  |
| Pathological stage, n (%) | 0 | 3 (3.0%) | 19 (11.7%) | .005 | 3 (8.1%) | 8 (17.4%) | .07 |
|  | IA1 | 32 (32.0%) | 72 (44.2%) |  | 9 (24.3%) | 18 (39.1%) |  |
|  | IA2 | 35 (35.0%) | 49 (30.1%) |  | 14 (37.8%) | 16 (34.8%) |  |
|  | IA3 | 5 (5.0%) | 6 (3.7%) |  | 4 (10.8%) | 1 (2.2%) |  |
|  | IB | 11 (11.0%) | 11 (6.7%) |  | 3 (8.1%) | 3 (6.5%) |  |
|  | IIB | 9 (9.0%) | 4 (2.5%) |  | 4 (10.8%) | 0 |  |
|  | IIIA | 5 (5.0%) | 2 (1.2%) |  | 0 | 0 |  |
| Histological type | Adenocarcinoma | 85 (85.0%) | 152 (93.3%) | .035 | 33 (89.2%) | 41 (89.1%) | 1 |
|  | Other | 15 (15.0) | 11 (6.7%) |  | 4 (10.8%) | 5 (10.9%) |  |
| Pleural invasion, n (%) | Yes | 14 (14.0%) | 12 (7.4%) | .09 | 5 (13.5%) | 2 (4.3) | .23 |
| Lymphovascular invasion, n (%) | Yes | 30 (30.0%) | 24 (14.7%) | .004 | 6 (16.2%) | 14 (30.4%) | .20 |

DLCO, diffusing lung capacity for carbon monoxide; FEV, forced expiratory volume; HA-ESM, height-adjusted erector spinae muscle; IQR, interquartile range; .

Supplementary Table 2. Baseline characteristics of patients in the lobectomy and segmentectomy groups after propensity score matching

| Factor | | |  | Lobectomy | | | | |  | | | Segmentectomy | | |  | | |
| --- | --- | --- | --- | --- | --- | --- | --- | --- | --- | --- | --- | --- | --- | --- | --- | --- | --- |
|  |  | | | | High HA–ESM | | Low HA–ESM | |  | | High HA–ESM | | | Low HA–ESM | | |  |
|  |  | | | | n=43 | | n=43 | | *P* | | n=41 | | | n=41 | | | *P* |
| Age, years | Median | | | | 69 | | 71 | | .88 | | 69 | | | 67 | | | .63 |
|  | IQR | | | | 63–76 | | 63–75 | |  | | 64–72 | | | 63–72 | | |  |
| Sex, n (%) | Male | | | | 23 (53.5%) | | 26 (60.5%) | | .66 | | 19 (46.3%) | | | 20 (48.8%) | | | 1 |
| Smoking status, n (%) | Smoker | | | | 23 (53.5%) | | 25 (58.1%) | | .83 | | 23 (56.1%) | | | 22 (53.7%) | | | 1 |
| FEV1, n (%) | ≥70 | | | | 34 (79.1%) | | 35 (81.4%) | | 1 | | 33 (80.5%) | | | 28 (68.3%) | | | .31 |
| Charlson Comorbidity Index | Median | | | | 2 | | 2 | | .44 | | 2 | | | 2 | | | .24 |
|  | IQR | | | | 2–3 | | 2–4 | |  | | 2–3 | | | 2–4 | | |  |
| Hypertension | Yes | | | | 15 (34.9%) | | 15 (34.9%) | | 1 | | 12 (29.3%) | | | 6 (14.6%) | | | .18 |
| Myocardial infarction or heart failure | Yes | | | | 3 (7.0%) | | 3 (7.0%) | | 1 | | 3 (7.3%) | | | 0(0%) | | | .24 |
| %FEV1 | Median | | | | 95.1 | | 96.8 | | .77 | | 94.7 | | | 93.8 | | | .91 |
|  | IQR | | | | 85.5–110 | | 85.1–110 | |  | | 84.8–111 | | | 84.8–101 | | |  |
| %DLCO | Median | | | | 80.8 | | 78.3 | | .40 | | 77.7 | | | 78.1 | | | .76 |
|  | IQR | | | | 70.6–92.7 | | 67.6–87.2 | |  | | 67.7–87.5 | | | 70.9–84.6 | | |  |
| Operation side, n (%) | Right | | | | 36 (83.7%) | | 34 (79.1%) | | .78 | | 20 (48.8%) | | | 15 (36.6%) | | | .37 |
|  | Left | | | | 7 (16.3%) | | 9 (20.9%) | |  | | 21 (51.2%) | | | 26 (63.4%) | | |  |
| Operation lobe, n (%) | Upper | | | | 24 (55.8%) | | 25 (58.1%) | | .96 | | 22 (53.7%) | | | 26 (63.4%) | | | .38 |
|  | lower | | | | 10 (23.3%) | | 8 (18.6%) | |  | | 17 (41.5%) | | | 15 (36.6%) | | |  |
|  | Middle | | | | 8 (18.6%) | | 9 (20.9%) | |  | | 0 (0%) | | | 0 (0%) | | |  |
|  | Bilobe | | | | 1 (2.3%) | | 1 (2.3%) | |  | | 2 (4.9%) | | | 0 (0%) | | |  |
| Deauville score | Median | | | | 2 | | 2 | | .57 | | 2 | | | 2 | | | .36 |
|  | IQR | | | | 2–3 | | 2–4 | |  | | 2–3 | | | 1–3 | | |  |
| Whole tumor size on CT (mm) | | 18 | | | | 18 | | .82 | | 15 | | | 15 | | | .15 | |
|  | IQR | | | | 14–23 | | 15–22 | |  | | 13–18 | | | 13–20 | | |  |
| Solid tumor size on CT (mm) | | 13 | | | | 14 | | .64 | | 10 | | | 10 | | | .12 | |
|  | IQR | | | | 9–17 | | 10–17 | |  | | 8–15 | | | 5–12 | | |  |
| Clinical stage, n (%) | IA1 | | | | 13 (30.2%) | | 11 (25.6%) | | .81 | | 22 (53.7%) | | | 23 (56.1%) | | | 1 |
|  | IA2 | | | | 30 (69.8%) | | 32 (74.4%) | |  | | 19 (46.3%) | | | 18 (43.9%) | | |  |
| Pathological size (mm) | Median | | | | 16 | | 19 | | .45 | | 15 | | | 15 | | | .23 |
|  | IQR | | | | 14–21 | | 15–25 | |  | | 12–18 | | | 13–20 | | |  |
| Pathological invasive size (mm) | Median | | | | 11 | | 15 | | .28 | | 12 | | | 10 | | | .51 |
|  | IQR | | | | 8–18 | | 10–19 | |  | | 7–15 | | | 6–13 | | |  |
| Pathological stage, n (%) | 0 | | | | 2 (4.7%) | | 3 (7.0%) | | .11 | | 2 (4.9%) | | | 8 (19.5%) | | | .15 |
|  | IA1 | | | | 19 (44.2%) | | 10 (23.3%) | |  | | 15 (36.6%) | | | 16 (39.0%) | | |  |
|  | IA2 | | | | 14 (32.6%) | | 18 (41.9%) | |  | | 18 (43.9%) | | | 14 (34.1%) | | |  |
|  | IA3 | | | | 0 (0%) | | 5 (11.6%) | |  | | 0 (0%) | | | 1 (2.4%) | | |  |
|  | IB | | | | 2 (4.7%) | | 2 (4.7%) | |  | | 5 (12.2%) | | | 2 (4.9%) | | |  |
|  | IIB | | | | 5 (11.6%) | | 5 (11.6%) | |  | | 1 (2.4%) | | | 0 (0%) | | |  |
|  | IIIA | | | | 1 (2.3%) | | 0 (0%) | |  | | 0 (0%) | | | 0 (0%) | | |  |
| Histological type | Adenocarcinoma | | | | 37 (86.0%) | | 38 (88.4%) | | 1 | | 33 (80.5%) | | | 37 (90.2%) | | | .35 |
|  | Other | | | | 6 (14.0%) | | 5 (11.6%) | |  | | 8 (19.5%) | | | 4 (9.8%) | | |  |
| Pleural invasion, n (%) | Yes | | | | 4 (9.3%) | | 4 (9.3%) | | 1 | | 6 (14.6%) | | | 1 (2.4%) | | | .11 |
| Lymphovascular invasion, n (%) | Yes | | | | 12 (27.9%) | | 8 (18.6%) | | .44 | | 7 (17.1%) | | | 11 (26.8%) | | | .42 |

DLCO, diffusing lung capacity for carbon monoxide; FEV, forced expiratory volume; HA-ESM, height-adjusted erector spinae muscle; IQR, interquartile range.
